# Hypercobalaminemia in Outpatient Practice

**DOI:** 10.3390/jcm15218301

**Published:** 2026-10-25

**Authors:** Osman Radhwi

**Affiliations:** 1 Hematology Department, Faculty of Medicine, King Abdulaziz University, Jeddah 21859, Saudi Arabia; oradhwi@kau.edu.sa; Tel.: +966-126408486 (ext. 17044); 2 Hematology Research Unit, King Fahd Medical Research Center, King Abdulaziz University, Jeddah 21859, Saudi Arabia

**Keywords:** hypercobalaminemia, vitamin B12, assay variability, haptocorrin, holotranscobalamin, macro B12, functional deficiency, outpatient pathway

## Abstract

Elevated serum vitamin B12 is common in clinical practice, but its meaning is often unclear. The result may reflect recent supplementation, analytical variation, increased concentrations of B12 binding proteins, liver or kidney disease, hematologic or solid malignancy, or a macro B12 complex. Total serum B12 also does not reliably establish intracellular B12 sufficiency. This focused narrative review summarizes the laboratory basis, major clinical associations, and practical outpatient evaluation of hypercobalaminemia. Because assays and reference intervals differ, elevation should first be defined against the local laboratory range and confirmed when the result is borderline or unexpected. A careful supplement history and a focused assessment with a complete blood count, liver tests, and renal function will explain many cases. Methylmalonic acid or homocysteine is most useful when symptoms or risk factors still raise concern for functional deficiency, rather than as a routine test in every patient with elevated B12. Macro B12 should be considered after common and clinically important causes have been assessed. We propose a cautious outpatient pathway that separates clear supplement-related elevation from persistent unexplained elevation and from elevation accompanied by clinical or laboratory abnormalities. This pathway reflects the authors’ interpretation of the available evidence and has not been prospectively validated. Elevated B12 should be treated as a clinical marker, not as a diagnosis and not as an automatic indication for broad imaging or bone marrow examination.

## 1. Introduction

Serum vitamin B12 (cobalamin) is usually requested to investigate deficiency. An unexpectedly high result therefore creates a different clinical problem. Surveys suggest that elevated B12 is often poorly understood and that clinicians use inconsistent approaches to its evaluation [1]. Earlier reviews proposed that unexplained elevation should prompt consideration of serious disease, but no widely accepted outpatient guideline has emerged [2].

The term hypercobalaminemia is used inconsistently. Some publications apply fixed cutoffs near 900 to 1000 pg/mL (about 664 to 738 pmol/L), while others use values above 600 to 700 pmol/L (about 813 to 949 pg/mL) [2,3]. These cut-offs are specific to the assays and populations in which they were reported. For this review, hypercobalaminemia means a total serum or plasma B12 concentration above the upper reference limit of the reporting laboratory. The absolute value, its persistence, and the clinical context remain more important than a single universal cutoff. Concentrations are given in pg/mL, with pmol/L in parentheses at first use, and 1 pg/mL is approximately 0.738 pmol/L.

The epidemiology of hypercobalaminemia has changed in recent decades due to the growing availability and use of high-dose B12 supplements, particularly among individuals concerned about aging, cognitive decline, or fatigue. Sublingual tablets and high-dose combination products can deliver many times the recommended dietary allowance of 2.4 mcg/day [4]. This makes supplement-induced hypercobalaminemia an increasingly common finding in outpatient practice.

A high total B12 concentration is not a diagnosis and does not establish B12 toxicity. It may be caused by supplementation, altered concentrations of transport proteins, reduced clearance, cellular injury, or analytical interference. It can also coexist with functional deficiency. The aim of this review is to provide a readable clinical synthesis and a cautious pathway for outpatient practice. The proposed pathway is intended to support judgment, not replace standard evaluation of abnormal blood counts, liver disease, kidney disease, or suspected malignancy.

### Literature Search and Selection

This focused narrative review was informed by searches of PubMed/MEDLINE and Europe PMC from database inception to 20 October 2026. Search terms included combinations of “vitamin B12,” “cobalamin,” “hypercobalaminemia,” “hypercobalaminaemia,” “elevated vitamin B12,” and “high vitamin B12,” together with terms related to malignancy, hematologic disease, liver disease, kidney disease, mortality, supplementation, assay variability, transcobalamin, haptocorrin, holotranscobalamin, methylmalonic acid (MMA), homocysteine, and macro B12. Titles and abstracts were reviewed for relevance to the clinical and laboratory questions addressed in this review, and full texts were examined when potentially relevant. Additional articles were identified from the reference lists of key publications. Priority was given to primary studies, systematic reviews, major clinical reviews, and studies directly addressing the investigation or interpretation of elevated vitamin B12. The conduct and reporting of this narrative review were considered with reference to the SANRA (Supplementary Materials) framework for narrative reviews [5].

## 2. What the Total B12 Assay Measures

Understanding hypercobalaminemia requires a grasp of B12 transport biochemistry and the distinction between total and biologically active B12. Vitamin B12 circulates bound to two main protein classes: haptocorrin (transcobalamin I and III) and transcobalamin II (TCII) [6]. Haptocorrin, a 60–70 kDa protein from the R-binder or cobalophilin family, carries approximately 80% of serum B12, but this fraction is unavailable for cellular uptake. Transcobalamin II, a 42–47 kDa protein, carries only around 20% of serum B12 but constitutes the biologically active fraction, delivering B12 to tissues through specific receptors. Only the B12-TCII complex (termed holotranscobalamin when bound) is metabolically active and accessible to cells [6].

Haptocorrin is produced primarily by neutrophils and stored in granulocyte secondary granules. As a result, any condition that drives myeloid proliferation causes marked haptocorrin elevation and a rise in total serum B12 despite normal or even reduced metabolically active B12 (holotranscobalamin). Transcobalamin II is produced primarily by hepatocytes, with secondary contributions from endothelial cells, monocytes, and intestinal epithelial cells [6].

Most routine assays report total circulating B12. Only a minority of circulating B12 is attached to transcobalamin and available for receptor-mediated cellular uptake. Most is bound to haptocorrin, a slower clearing carrier that does not directly deliver B12 to most tissues [6,7]. As a result, a high total B12 concentration may reflect increased carrier protein rather than increased biologically available vitamin B12.

This distinction explains several apparently paradoxical findings. Myeloid proliferation may raise haptocorrin and total B12. Liver injury may release stored B12 and binding proteins. Kidney dysfunction may alter clearance and carrier metabolism. A patient can therefore have high total B12 while tissue availability remains uncertain [6,8,9]. Clinical symptoms and functional markers should be considered when deficiency remains plausible.

## 3. Assay Variability and Confirmation

**Figure 1:**
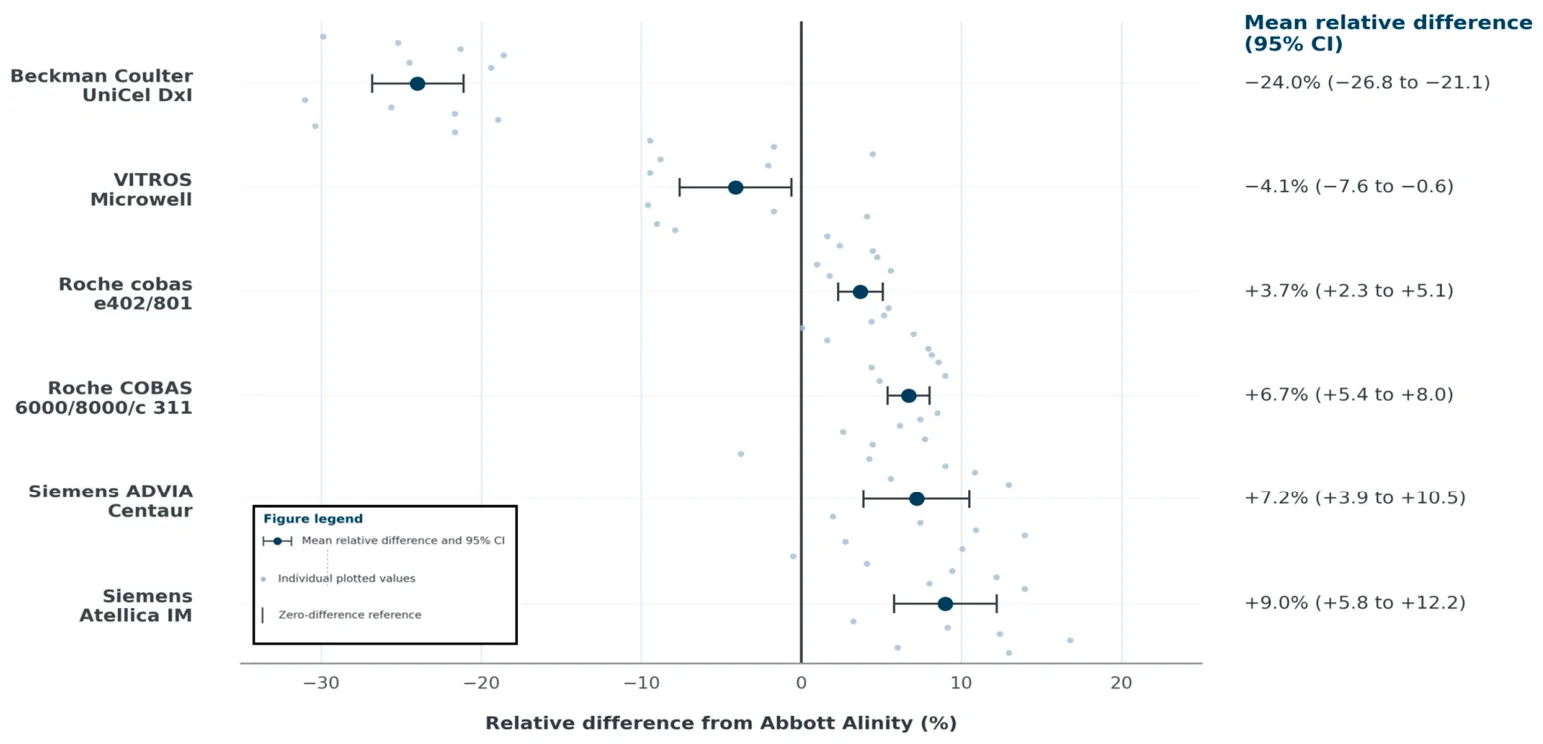
Mean relative differences from Abbott Alinity for six automated vitamin B12 immunoassays. Data were derived from 12 paired external quality assurance samples reported in Table 2 of Seelan et al. (2025) [3]. Small circles represent individual samples, large circles indicate mean paired relative differences, and horizontal bars represent 95% confidence intervals calculated using Student’s *t* distribution (*n* = 12). The vertical line at 0% indicates no difference from Abbott Alinity. Positive (+) and negative (−) values indicate higher and lower measurements relative to Abbott Alinity, respectively. Relative difference (%) = [(platform − Abbott)/Abbott] × 100. The figure was replotted from the published tabulated data rather than reproduced from the original graphic. Abbreviations: CI, confidence interval

Total B12 assays are method dependent. Automated platforms differ in sample pre-treatment, binding reagents, calibration, and signal detection. Standardization efforts exist, including the WHO International Standard 03/178 [10], but complete numerical harmonization between platforms has not been achieved. In an external quality assurance comparison, Seelan et al. (2025) measured 12 samples on six platforms against Abbott Alinity [3]. Mean relative bias was −24.0% for Beckman Coulter UniCel DxI (95% CI −26.8 to −21.1%), −4.1% for VITROS Microwell (−7.6 to −0.6%), +3.7% for Roche cobas e402/801 (+2.3 to +5.1%), +6.7% for Roche COBAS 6000/8000/c 311 (+5.4 to +8.0%), +7.2% for Siemens ADVIA Centaur (+3.9 to +10.5%), and +9.0% for Siemens Atellica IM (+5.8 to +12.2%). Absolute differences varied in direction as well as size: Beckman Coulter values were lower than Abbott in every sample (−120 to −192 pg/mL), whereas Siemens differences ranged from −15 to +169 pg/mL. Correlation with Abbott was high on every platform (R^2^ 0.965 to 0.9998) [3]. These were external quality assurance samples rather than patient sera, so the comparison describes analytical agreement and does not establish clinical concordance between platforms. Despite this agreement, a value near the upper reference limit may move from normal to elevated when measured on another platform (Figure 1).

Cross-platform testing can be useful when analytical interference is suspected, but it should not be used as if one routine platform were a definitive reference method. A repeat test is less important when the value is markedly high and the clinical explanation is already clear, such as soon after an injection. Laboratory reports should include the analyzer platform name to enable clinicians to contextualize results appropriately and distinguish between analytical variation and true biologic change [3]. Additionally, analytical interference should be considered when the measured B12 concentration is markedly discordant with the clinical picture. Reported mechanisms include heterophile antibodies, anti-intrinsic factor antibodies in susceptible assay systems, and biotin interference in immunoassays that use streptavidin biotin technology. In such cases, discussion with the laboratory and confirmation using an alternative analytical method or assay platform may be appropriate [11,12].

In practice, serial monitoring should use the same analyzer where possible. When a laboratory changes platforms, new results should be compared with previous ones using the laboratory’s own method comparison rather than assuming equivalence. Seelan et al. provide regression equations that could in principle convert results between analyzers [3]; these were not validated for clinical use in this review and should be verified locally before use. Reports should state the analyzer name and the reference interval used [3].

## 4. Clinical Significance and Prognosis

The clinical meaning of elevated B12 depends heavily on the population. In hospitalized older adults, elevated B12 has been associated with higher short-term mortality. In one internal medicine cohort, one-year mortality was 39% among patients with B12 above 600 pmol/L (813 pg/mL) as measured in that cohort’s assay and 26% among those with normal B12. The adjusted hazard ratio was 1.71 [13]. This association should be interpreted as a marker of illness burden rather than proof that B12 itself causes harm.

Most prognostic data are derived from hospitalized and older adults, and outpatient or community outcome data are limited. A meta-analysis of cohort studies, including population-based cohorts, found a dose-dependent association. Each 136 pg/mL (100 pmol/L) increase in serum B12 was associated with a 4% higher risk of all-cause mortality in the general population (HR 1.04, 95% CI 1.01–1.08) and a 6% higher risk in older adults (HR 1.06, 95% CI 1.01–1.13). B12 above 813 pg/mL (600 pmol/L) was associated with 50% higher all-cause mortality (HR 1.50, 95% CI 1.29–1.74) and a non-significant trend toward higher cardiovascular mortality (HR 2.04, 95% CI 0.99–4.19; two studies) [14]. Community data on cancer incidence come from a Danish population cohort [15]. These findings highlight the importance of context-sensitive interpretation: a B12 level of 1200 pg/mL (886 pmol/L) in an asymptomatic young person taking supplements may be of little clinical consequence in itself, whereas the same value in an elderly hospitalized patient with cytopenias, weight loss, and hepatomegaly warrants urgent evaluation

## 5. Causes of Hypercobalaminemia

The differential diagnosis is best organized by mechanism and clinical context. More than one mechanism may be present in the same patient (Table 1).

### 5.1. Non-Pathological Causes: Supplementation

Exogenous B12 is the most direct explanation for a high result. B12 is water-soluble, but, unlike most water-soluble vitamins, it is stored substantially in the liver [6,9], so the body can retain it for prolonged periods. The bioavailability of supplemental vitamin B12 varies according to route and formulation. In addition to intrinsic factor-dependent absorption in the terminal ileum, a small proportion of free vitamin B12 is absorbed through passive diffusion independent of intrinsic factors. Estimates of passive absorption vary but are commonly around 1%, which becomes clinically relevant when oral doses of several hundred to several thousand micrograms are administered [6,8]. Sublingual lozenges and liquid formulations attempt to maximize absorption through oral mucosa, though evidence for superior bioavailability compared to swallowed tablets is limited. Intramuscular injections bypass absorption entirely, delivering 1000 mcg of cyanocobalamin in a single dose that raises serum B12 acutely.

It is worth distinguishing between cyanocobalamin (the synthetic form most frequently used in supplements and low-cost preparations) and methylcobalamin (the active form, marketed as “natural” or more bioavailable). Both forms raise serum B12. Evidence that methylcobalamin yields higher serum B12 than cyanocobalamin is limited, and this review does not recommend one form over the other.

History should include prescribed injections, tablets, sublingual preparations, multivitamins, and fortified products. Patients often report only prescription medicines unless asked specifically. The measured concentration depends on dose, route, adherence, timing of the last dose, renal function, and the assay. The literature does not establish a single washout period that applies to all preparations.

#### Post-Supplementation Kinetics and the Repeat Test

Time to normalization of serum vitamin B12 after supplementation is variable and influenced by route, formulation, dose, duration of use, renal function, and other patient-specific factors. After parenteral cyanocobalamin, a substantial proportion of the dose is excreted in urine, and retention varies between individuals [16]. Therefore, no universal time frame for normalization can be applied. In our view, when confirmation of a supplement-related elevation would change management, repeat measurement after approximately 8 to 12 weeks of cessation is a practical approach. This interval represents expert opinion rather than a validated cutoff. Patients who require lifelong replacement should not stop treatment merely to normalize the laboratory value. Persistent elevation after confirmed cessation should prompt evaluation through the unexplained elevation pathway.

Total body B12 stores in healthy adults are commonly estimated at 2 to 3 mg [4]. Assuming no absorption and intact enterohepatic circulation, the IOM estimates that stores of 1 mg, 2 mg, and 3 mg would meet the body’s needs for about 3, 5, and 6 years, respectively [4]. These are estimates of the time for stores to be depleted, not of the time for serum B12 to normalize after supplementation.

Long-term loss of body vitamin B12 can be approximated by an exponential process, and kinetic studies have reported daily losses of approximately 0.1% of the body pool in individuals with intact absorption and enterohepatic recycling, although estimates vary substantially between individuals. Under a constant fractional loss model of 0.1%/day, the corresponding mathematical half-life is approximately 693 days, or 1.9 years. This calculated value describes turnover of body stores and should not be interpreted as the time required for serum vitamin B12 to normalize after supplementation is discontinued [4,17].

### 5.2. Hematologic Disease

High B12 is well-described in chronic myeloid leukemia (CML), polycythemia vera (PV), myelofibrosis (MF), hypereosinophilic disorders, and other myeloproliferative neoplasms (MPN). Increased production of haptocorrin released by proliferating myeloid cells is the main proposed mechanism [6,7]. Elevated B12 is not a diagnostic criterion for these diseases. Its value lies in prompting attention to the CBC, differential, blood film, and physical examination when the result is otherwise unexplained.

The threshold for hematology referral should be determined by the accompanying findings. Cytopenias, leukocytosis, thrombocytosis, erythrocytosis, basophilia, eosinophilia, immature cells, dysplasia, splenomegaly, or constitutional symptoms are meaningful triggers. Mild isolated elevation with a normal CBC and no clinical concern is not, by itself, an indication for serum tryptase, molecular testing, or bone marrow biopsy.

### 5.3. Liver and Kidney Diseases

Liver disease is a frequent association. The liver is the main storage organ for B12, containing approximately half of total body B12 stores [4]. Hepatocellular injury may release stored B12 into circulation, and reduced hepatic uptake of haptocorrin-bound B12 and reduced hepatic production of transcobalamin II may further increase the circulating concentration [6,14].

Kidney dysfunction is also associated with elevated serum B12, likely through altered renal handling and changes in circulating B12 binding proteins. Both holotranscobalamin I and holotranscobalamin II have been reported to increase in renal failure, suggesting that several mechanisms may contribute [18]. Moreover, serum B12 does not show a consistent linear relationship with renal function, supporting the view that hypercobalaminemia in kidney disease cannot be explained by reduced clearance alone [19]. In this setting, the underlying renal disorder should be evaluated and managed through its usual clinical pathway.

### 5.4. Solid Malignancy Associations

Elevated B12 has been associated with solid tumors in observational studies, but the finding is nonspecific and does not establish causality [6,7]. Population-based data also support an association between markedly elevated B12 and subsequently diagnosed cancer. In a Danish cohort, plasma B12 above 1084 pg/mL (800 pmol/L) was associated with a substantially increased incidence of cancer during the first year after measurement, with the strongest association occurring soon after testing [15]. This elevation may reflect tumor burden, host inflammatory response, liver involvement, or general illness. The result should strengthen attention to symptoms, examination, age-appropriate screening, and other abnormal tests. Current evidence does not justify routine whole-body imaging for an isolated high B12 result in an otherwise well patient.

## 6. Functional Deficiency and Macro B12

A normal or high total B12 concentration does not exclude deficiency. Assay limitations, altered binding proteins, and impaired cellular delivery can obscure the relationship between total B12 and tissue status [8,9]. In patients with compatible neurologic or hematologic findings, MMA is generally more specific than homocysteine for B12-related metabolic impairment. Kidney dysfunction can raise MMA independently of B12 status, and homocysteine is influenced by folate status and other conditions [8,20].

MMA and homocysteine should therefore not be ordered automatically for every elevated B12 result. They are most useful when the clinical picture still suggests deficiency, when the total B12 result conflicts with symptoms, or when a borderline result is difficult to interpret. Strong clinical suspicion should not be dismissed solely because total B12 is normal or high. Two hypothetical scenarios illustrating this approach are summarized in Box 1.

Box 1 **Hypothetical illustrative clinical scenarios.**

Case A. A 45-year-old woman takes sublingual cyanocobalamin 1000 mcg daily for fatigue. Total B12 is 1400 pg/mL. CBC, liver tests, and eGFR are normal, and there are no neurological symptoms.

Management: stop the supplement if safe and repeat after 8 to 12 weeks only if the result will change management. MMA is not required.

Case B. A 70-year-old man has taken metformin for 10 years and a proton pump inhibitor. He reports symmetrical distal paresthesia and unsteady gait. Total B12 is 1100 pg/mL. MMA is elevated and interpreted in light of eGFR.

Management: follow the clinical probability of deficiency rather than the total B12 result.

Macro B12 describes a high-molecular-weight complex involving B12, a binding protein, and immunoglobulin. Reduced clearance can produce persistent high total B12. In a recent selected cohort with B12 above 2000 pg/mL (1476 pmol/L), PEG precipitation supported macro B12 in 24% out of 72 individuals. One patient also had elevated MMA, showing that macro B12 does not exclude functional deficiency [21]. Case-based laboratory studies also show that defining the exact complex is technically difficult and assay-dependent [22].

PEG precipitation is a supportive laboratory test, not a confirmatory one. Precipitation is non-specific: it can co-precipitate other proteins, recovery depends on the protocol and the laboratory, and the result depends on the cut-off chosen [21,22]. A positive result supports, but does not confirm, a macro B12 complex, and a negative result does not exclude one. PEG precipitation should therefore be used only when the focused evaluation is unrevealing and the probability of serious disease is low, and the test should be validated in the laboratory that performs it.

There is currently no universally accepted method or cutoff for identifying macro B12 in routine clinical practice [21]. PEG precipitation has been used as a practical screening method, but it is nonspecific, and recovery may vary according to analytical conditions [23]. More specific approaches, including measurement of B12 binding proteins and size exclusion chromatography, are largely confined to specialized laboratories [23]. Further work is needed to define and validate clinically useful approaches for identifying macro B12 [21,23].

Historically, unsaturated cobalamin binding capacity (UBBC) was used to characterize abnormalities in circulating B12 binding proteins. In a study of 228 reference individuals and 277 hospitalized patients, Gimsing and Nexø established a plasma UBBC reference interval of 500 to 1200 pmol/L and showed that increased unsaturated haptocorrin was particularly associated with MPNs and acute nonlymphatic leukemia, whereas increased unsaturated transcobalamin was observed in some lymphoproliferative and autoimmune disorders [24]. More recent laboratory reviews favor direct measurement of haptocorrin and transcobalamin when further characterization of unexplained elevated B12 is required. UBBC has not been prospectively validated as a routine test in the outpatient evaluation of hypercobalaminemia and should therefore be regarded as a specialized or historical investigation rather than a standard second line test [24].

## 7. Outpatient Management: Diagnostic Algorithm and Referral Criteria

**Figure 2:**
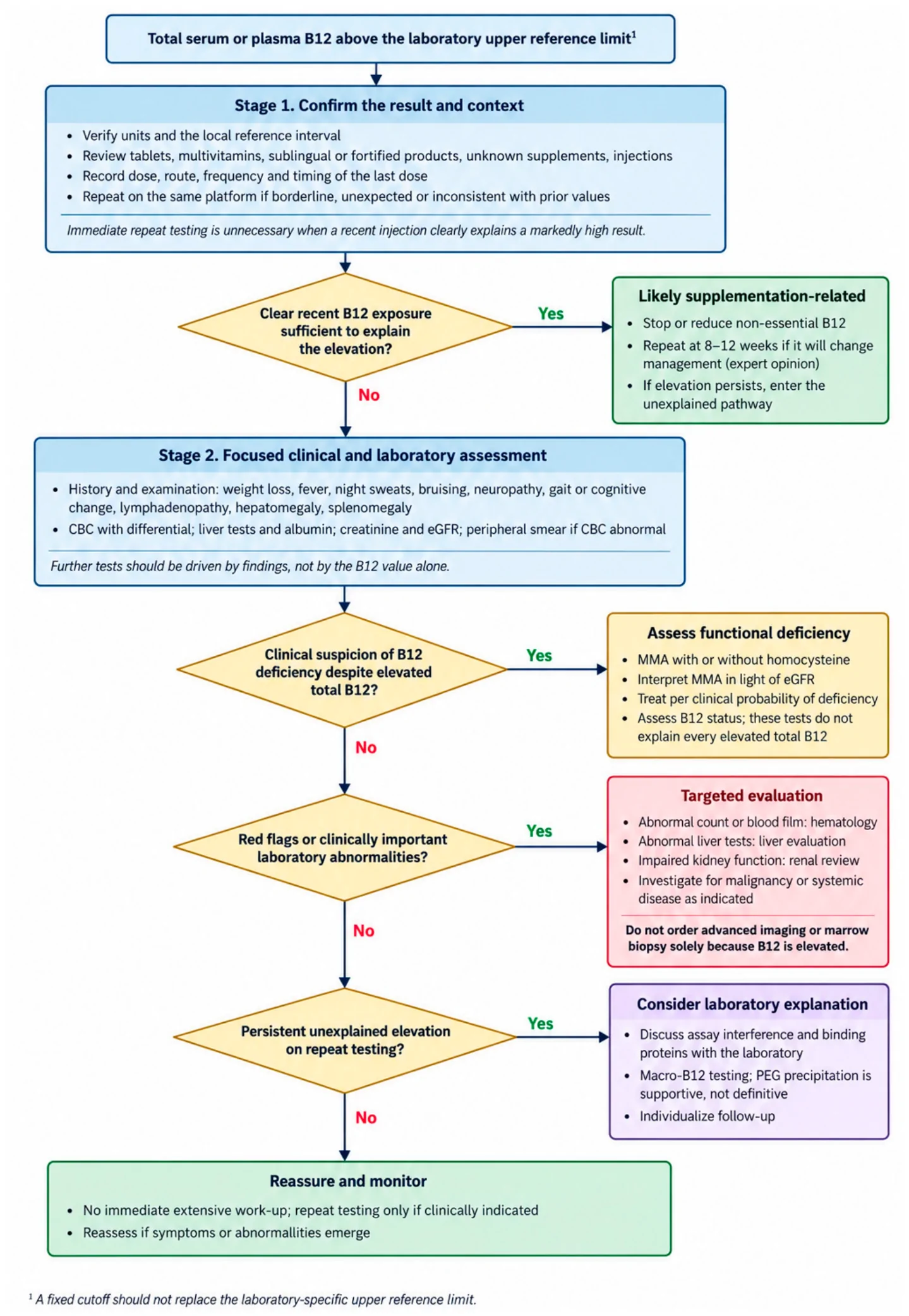
Author-proposed outpatient pathway for elevated total vitamin B12. The pathway is a narrative synthesis and expert opinion and has not been prospectively validated. Thresholds refer to the laboratory upper reference limit and are not universal values. Abbreviations: CBC, complete blood count; eGFR, estimated glomerular filtration rate; MMA, methylmalonic acid; PEG, polyethylene glycol.

Managing hypercobalaminemia in the outpatient setting begins with a thorough history and physical examination aimed at identifying the underlying cause and determining the need for further evaluation. The algorithm below (Figure 2) provides a structured approach. This algorithm has not been prospectively validated. Its diagnostic accuracy, safety, and effect on unnecessary testing should be assessed in prospective studies before it is adopted as guidance.

## 8. Special Populations

### 8.1. Children

The pediatric evidence is limited and should not be extrapolated directly from adults. In a retrospective study of 388 children with very high B12, 45.4% had received B12 treatment and 81.3% were asymptomatic. Five children without a known chronic disease or supplementation history were later diagnosed with malignancy [25]. The finding supports follow-up of persistent unexplained elevation, but it does not establish elevated B12 as a cancer screening test.

Elevated B12 was also more frequent in a pediatric neurology cohort than in a general pediatric clinic, with epilepsy common among those with persistent elevation. The retrospective design does not establish that B12 contributes to neurologic disease [26]. In children with cystic fibrosis and pancreatic insufficiency, high supplement intake strongly predicted high serum B12, showing how disease-specific supplementation can dominate interpretation [27].

### 8.2. Patients with Neurologic Symptoms

B12 deficiency can present with neuropathy or myelopathy without anemia, and neurologic recovery may be incomplete when treatment is delayed [8,28]. A high total B12 result should therefore not end the assessment when the clinical pattern remains compatible with deficiency. MMA, homocysteine, holotranscobalamin where available, and a therapeutic trial may each have a role, but none should replace the clinical examination [8,9,28].

## 9. Limitations

The evidence base is heterogeneous. Most disease associations come from retrospective or cross-sectional studies, and confounding by illness severity is substantial. Cutoffs differ across assays and populations. Studies of macro B12 are small, and laboratory methods are not standardized. Data supporting a specific supplement washout period, a universal referral threshold, or a cancer screening strategy are insufficient. The proposed outpatient pathway has not been prospectively tested and should be evaluated in clinical cohorts before it is presented as a guideline.

Prognostic evidence is derived largely from hospitalized and older populations, and outpatient outcome data are limited. The proposed pathway has not been prospectively validated, and prospective diagnostic accuracy and implementation studies are needed.

## 10. Conclusions

Elevated total B12 is common, but it is not self-explanatory. The first task is to verify the assay context and identify supplementation. Persistent unexplained elevation should prompt a focused review of the CBC, liver function, kidney function, and clinical findings. Functional markers are reserved for patients in whom deficiency remains plausible. Macro B12 is a useful later consideration when the focused evaluation is reassuring. The result should guide attention, not trigger indiscriminate testing. A readable, risk-based outpatient approach can identify patients who need targeted evaluation while protecting low-risk patients from unnecessary imaging and invasive procedures.

## Figures and Tables

**Table 1:** Causes of hypercobalaminemia by mechanism, with clinical clues and a practical first step.

Cause	Likely Mechanism	Clinical Clues	Practical First Step
Supplementation	Recent oral, sublingual, or injected B12 increases circulating B12.	Prescribed injections, high-dose tablets, multivitamins, fortified products.	Document dose, route, and timing. Stop nonessential products when safe; repeat only if the result affects management.
Myeloid and other hematologic disorders	Increased haptocorrin from proliferating myeloid cells; some lymphoid disorders may alter transcobalamin.	Abnormal CBC, leukocytosis, eosinophilia, cytopenias, abnormal smear, splenomegaly, constitutional symptoms.	Follow abnormal blood findings through the usual hematologic pathway. B12 alone is not an indication for marrow examination.
Liver disease	Release of stored B12 and binding proteins from injured hepatocytes; impaired clearance.	Abnormal liver tests, alcohol-related disease, hepatitis, cirrhosis, hepatomegaly.	Liver tests and clinical assessment; image or refer per standard liver indications.
Kidney disease	Reduced clearance and altered metabolism of binding proteins.	Reduced eGFR, proteinuria, edema, known CKD.	Interpret B12 and MMA in the context of renal function; refer per the renal disorder.
Solid malignancy and systemic illness	Tumor burden, inflammation, tissue injury, and altered binding proteins may contribute.	Weight loss, focal symptoms, abnormal examination or imaging.	Age-appropriate screening and symptom-directed investigation. Routine pan-cancer imaging is not supported for isolated elevation.
Macro B12 or analytical interference	B12-containing immunoglobulin complexes or assay-dependent interference.	Persistent unexplained elevation with stable clinical state and unrevealing focused evaluation.	Discuss with the laboratory; PEG precipitation is supportive, not definitive.
Functional B12 deficiency despite normal or high total B12	Total B12 may not reflect intracellular delivery or use.	Neuropathy, myelopathy, cognitive symptoms, macrocytosis, malabsorption risk.	Measure MMA ± homocysteine when clinically indicated; account for renal function; do not delay treatment in a convincing symptomatic case.

CBC, complete blood count; CKD, chronic kidney disease; eGFR, estimated glomerular filtration rate; MMA, methylmalonic acid; PEG, polyethylene glycol.

## Data Availability

No new data were created or analyzed in this study. Data sharing is not applicable to this article.

## Supplementary Materials

The following supporting information can be downloaded at: https://www.mdpi.com/article/10.3390/jcm15218301/s1, Table S1: Total SANRA self-assessment score: 11/12.

## Abbreviations

B12, vitamin B12 (cobalamin); CBC, complete blood count; CI, confidence interval; CKD, chronic kidney disease; eGFR, estimated glomerular filtration rate; EQA, external quality assurance; MMA, methylmalonic acid; MPN, myeloproliferative neoplasm; PEG, polyethylene glycol; TCII, transcobalamin II; UBBC, unsaturated B12-binding capacity; pg/mL, picograms per millilitre; pmol/L, picomoles per litre.
