# Supplementary material for: Hypercobalaminemia in Outpatient Practice
Source: J Clin Med. 2026 Oct 25;15(21):8301. doi: 10.3390/jcm15218301 (PMC13764272; doi:10.3390/jcm15218301)
Supplement: Supplementary file 1 [file jcm-15-08301-s001.zip › jcm-4599949-supplementary.pdf]

Supplementary Material S1. SANRA self-assessment

Manuscript: Hypercobalaminemia in Outpatient Practice

The manuscript was self-assessed using SANRA, the Scale for the Assessment of Narrative Review Articles. SANRA contains six items scored from 0 to 2, for a maximum score of 12. This self-assessment is intended as a reporting-quality check and does not constitute external validation of the review or the proposed clinical pathway.

Item	SANRA criterion	Score	Rationale and manuscript location
1	Justification of the article's importance for the readership	2	The Introduction identifies elevated B12 as a common but inconsistently interpreted outpatient finding and explains the clinical consequences of both under-investigation and unnecessary testing.
2	Statement of concrete aims or formulation of questions	2	The Introduction states the aim of providing a readable clinical synthesis and a cautious outpatient pathway for interpreting elevated total B12.
3	Description of the literature search	2	The Literature search and selection subsection reports the databases and supplementary retrieval source, search date range, core and targeted search terms, eligibility criteria, evidence prioritization, hand-searching, and the purposive narrative selection approach.
4	Referencing	2	Key mechanistic, analytical, epidemiologic and clinical statements are referenced. Statements based primarily on author judgment are identified as practical proposals or expert opinion.
5	Scientific reasoning	2	The review distinguishes association from causation, separates analytical and biological mechanisms, gives greater weight to systematic reviews and primary studies when available, and explicitly discusses uncertainty and limitations.
6	Appropriate presentation of data	1	Relevant denominators, event rates, hazard ratios and selected quantitative findings are presented where they materially inform interpretation. Because the underlying evidence is heterogeneous and the review is narrative, quantitative effect estimates are not available or summarized consistently for every association.

Supplementary Table 1. Total SANRA self-assessment score: 11/12.

Reference

Baethge C, Goldbeck-Wood S, Mertens S. SANRA- a scale for the quality assessment of narrative review articles. Research Integrity and Peer Review. 2019;4:5. doi:10.1186/s41073-019-0064-8.
